# Prevalence of Human Coronaviruses in Children and Phylogenetic Analysis of HCoV-OC43 during 2016–2022 in Riyadh, Saudi Arabia

**DOI:** 10.3390/v14122592

**Published:** 2022-11-22

**Authors:** Khalid A. Alamri, Mohamed A. Farrag, Ibrahim M. Aziz, Gani Asa Dudin, Arif Ahmed Mohammed, Fahad N. Almajhdi

**Affiliations:** 1 Department of Botany and Microbiology, College of Science, King Saud University, Riyadh 11451, Saudi Arabia; 2 Center of Excellence in Biotechnology Research, King Saud University, Riyadh 11451, Saudi Arabia

**Keywords:** OC43, NL63, 229E, HKU1, human coronaviruses, sequence and phylogenetic analysis, epidemiology

## Abstract

With the emergence of SARS-CoV-2, routine surveillance combined with sequence and phylogenetic analysis of coronaviruses is urgently required. In the current study, the four common human coronaviruses (HCoVs), OC43, NL63, HKU1, and 229E, were screened in 361 clinical samples collected from hospitalized children with respiratory symptoms during four winter seasons. RT-PCR-based detection and typing revealed different prevalence rates of HCoVs across the four seasons. Interestingly, none of the four HCoVs were detected in the samples (*n* = 100) collected during the winter season of the COVID-19 pandemic. HCoV-OC43 (4.15%) was the most frequently detected, followed by 229E (1.1%). Partial sequences of S and N genes of OC43 from the winter seasons of 2015/2016 and 2021/2022 were used for sequence and phylogenetic analysis. Multiple sequence alignment of the two Saudi OC43s strains with international strains revealed the presence of sequence deletions and several mutations, of which some changed their corresponding amino acids. Glycosylation profiles revealed a number of O-and N-glycosylation sites in both genes. Based on phylogenetic analysis, four genotypes were observed with Riyadh strains grouped into the genotype C. Further long-term surveillance with a large number of clinical samples and sequences is necessary to resolve the circulation patterns and evolutionary kinetics of OC43 in Saudi Arabia.

## 1. Introduction

Coronaviruses are globally distributed and infect a wide range of hosts including humans. CoVs cause systemic infections where several organs are attacked, including the respiratory tract, gastrointestinal tract, kidneys, liver, and nervous system [1,2,3]. In the case of human CoVs, the symptoms range from insignificant, self-limiting infection of the upper respiratory tract (i.e., common cold) to severe and even fatal infections that are associated with pneumonia, renal failure, liver injury, and septic shock [4,5,6,7,8]. With the identification of SARS-CoV-2, seven human CoVs are now known to infect the human population: HCoV-229E, HCoV-OC43, HCoV-NL63, HCoV-HKU1, SARS-CoV-1, MERS-CoV, and SARS-CoV-2. The first four types usually cause the common cold with mild and self-limiting infection of the upper respiratory tract in immunocompetent individuals [9]. The remaining three types are the causative agents of CoV outbreaks/pandemics in the last two decades [4,10]. The four common HCoVs, OC43, NL63, 229E, and HKU1, show differences in the frequency of detection in different countries at different times. In general, OC43 is the most frequently detected, followed by NL63, and HKU1 and 229E have the lowest detection frequency [11,12,13,14].

According to the latest International Committee on the Taxonomy of Viruses (ICTV) report, HCoVs are grouped within the subfamily Orthocoronavirinae, family Coronaviridae, and order Nidovirales. CoV particles are enveloped with three structural proteins that decorate the viral envelope; envelop (E), membrane (M), and spike protein (S). The S protein constitutes long projections and gives the virus its characteristic crown-like structure or corona shape. Orthocoronavirinae have four genera: α-coronavirus (229E and NL63), β-coronavirus (OC43, HKU1, SARS-CoV-1, MERS-CoV, and SARS-CoV-2), γ-coronavirus (*Avian coronavirus*), and δ-coronavirus (*Coronavirus HKU15*) [1,7,15]. The latter two genera do not infect humans. CoVs genome is a non-segmented, positive sense, single-stranded RNA genome. It is the longest (26.4–32.0 kb) among all known RNA viruses [16,17,18]. The genome is flanked by two untranslated regions (UTRs) and was predicted to have around 14 ORFs with more than 25 proteins. The first two ORFs, ORF1ab and ORF1a, occupy approximately three-fourths of the genome size and code for 16 non-structural proteins. Non-structural proteins are mainly involved in virus replication where they serve several functions such as RNA-dependent RNA polymerase (nsp12), helicase (nsp13), protease (nsp5), and papain-like protease (nsp3) [19]. Structural proteins (E, M, N, and S) along with a number of accessory proteins are encoded by the last third ORFs [20,21].

Viruses with RNA genomes including CoVs are known to form viral quasispecies because of the polymerase errors during virus replication [22]. The formation of quasispecies enables viruses to evade pre-existing immunity and to adapt to a variety of environments. The large genome of CoVs allows these viruses to evolve through genetic recombination besides point mutations. In this way, CoVs are frequently evolving, crossing species boundaries and adapting to rapidly changing niches [23].

Due to their high morbidity and mortality rates, the three SARS-CoV-1, SARS-COV-2, and MERS-CoV received much attention regarding their epidemiology and evolutionary kinetics. On the other hand, the epidemiologies and genomic characteristics of the four common HCoVs are poorly described in the literature. Therefore, performing sequence and phylogenetic analysis of HCoVs will help to predict, prevent, and control any possible HCoV outbreaks. In Saudi Arabia, more than 10 million Muslims from around 184 different countries with different ethnicities and socioeconomic backgrounds are gathered in the holy places during the Hajj and Umrah seasons. In addition, over 11 million foreign workers from more than 100 countries are in a dynamic movement back and forth between their home countries and Saudi Arabia. In such conditions, new viral strains can be introduced into Saudi Arabia and can be spread to other countries [24]. Despite this situation, there is a lack of knowledge regarding the sequence and phylogenetic analysis of the four human CoVs. Most of the studies are mainly focused on virus detection and clinical outcomes. Therefore, in the current study, the prevalence of the four HCoVs was investigated. RT-PCR assay was used to detect and type HCoVs in clinical samples collected from two hospitals in Riyadh. Sequence and phylogenetic analysis of S and N genes of OC43 were performed to determine the genotype of circulating Saudi stains.

## 2. Materials and Methods

### 2.1. Clinical Samples and Ethics Statement

A total of 361 nasopharyngeal aspirates (NPA) samples were collected from children hospitalized at King Khalid University Hospital (KKUH) and King Abdulla University Hospital (KAUH) during four winter seasons, 2014/2015, 2015/2016, 2019/2020, and 2021/2022. NPAs were collected from children displaying acute respiratory symptoms including rhinorrhea, cough, dyspnea, fever, and sneezing. The samples were obtained following the protocols approved by the Ethical Committee of King Saud University and after obtaining written informed consent from the parents/guardians of the patients. The collected NPAs were mixed immediately with 2 mL of the viral transport medium (MEM supplemented with 500 units of penicillin and 0.5 mg streptomycin). Upon collection, samples were transported in an ice box to the Virology Research Laboratory (College of Science, King Saud University, Riyadh, Saudi Arabia) for processing, aliquoted, and stored at −80 °C till use.

### 2.2. Detection and Typing of HCoVs

Viral RNA was extracted from NPAs using QIAamp viral RNA extraction kit (Qiagen, Hilden, Germany) following the instructions of the company. RNAs were used as a template to screen the samples for the presence of the four HCoVs; NL63, OC43, HKU1, and 229E using the OneStep RT-PCR kit (Qiagen). Typically, for each 25 µL reaction, the following reagents were mixed; nuclease-free water 9 μL, OneStep RT-PCR buffer (5×) 5μL, dNTP mix (10 mM, each) 1 μL, forward primer (panCoV-F) 1.5 μL, reverse primer (panCoV-R) 1.5 μL (Table 1), RNase inhibitor 1 μL, OneStep RT-PCR enzyme mix 1 μL and 5 μL of the extracted RNA. The tubes were placed in the Gene-Amp 9700 thermal cycler (Applied Biosystems, Foster City, CA, USA) using the following cycling protocol: reverse transcription at 50 °C for 30 min, 1 cycle of initial PCR activation at 95 °C for 15 min, 35 cycles of denaturation at 94 °C for 30 s, primer annealing at 52 °C for 90 s, extension at 72 °C for 90 s, and final extension at 72 °C for 10 min. Typing reactions of positive samples were performed using the same conditions of detection and primer sets listed in Table 1. PCR products were visualized in 1% ethidium-bromide-stained agarose gel and compared to a DNA ladder (GelPilot 100 bp plus, cat. No. 239045; Qiagen).

### 2.3. Generation of Sequence Fragments of S and N Genes

RNAs of HCoV-OC43-positive samples were used to amplify sequence fragments of S (1052 nt) and N (837 nt) genes. Generation of the sequencing fragments was achieved as described in our previous study (Farrag et al., 2021) using SuperScript^®^ III One-Step RT-PCR System (Life Technologies, Carlsbad, CA, USA) and sequencing primers in Table 1. The cycling program involved one cycle at 55 °C for 30 min, one cycle at 94 °C for 2 min, 40 cycles at 94 °C for 15 s, 55 °C (S gene) and 57 °C (N gene) for 30 s and 68 °C for 1 min, and one cycle at 68 °C for 10 min. To remove impurities of PCR products, S and N fragments were purified using a QIAquick PCR purification kit (Qiagen) and were sequenced on both strands using BigDye Terminator version 3.1 sequencing kit on ABI PRISM 3730xl genetic analyzer at GATC Biotech (Cologne, Germany). The raw sequence data of both genes were edited and assembled using Bioedit software, version 7.2.5 (Ibis Biosciences, Carlsbad, CA, USA). The sequence of both genes was deposited in the gene bank database under the accession number (S gene: OP712601 and OP712602 and N gene: OP712603 and OP712604).

### 2.4. Sequence and Phylogenetic Analysis

A total of 59 (S gene) and 46 (N gene) HCoV-OC43 strains were retrieved from the GenBank database. The strains were selected to represent the different virus genotypes, different countries worldwide, and different years. Sequence fragments corresponding to S and N genes were edited and trimmed from the international sequences using the Editseq program of Lasergene software, version 3.18 (DNAStar, Madison, WI, USA). The prototype strain HCoV-OC43 (accession number: AY391777) isolated during the year 1967 was set as the reference strain. Sequences of Bovine CoVs isolated from France, Canada, and Japan were added to the alignment to root the trees [27]. Multiple sequence alignment for S and N genes and their corresponding amino acid sequences were generated and identification of mutation sites was performed using the Clustal W algorithm, MegAlign program, Lasergene v3.18. Heterogeneity in the glycosylation profiles of all stains was assessed by determining the potential N- and O-linked glycosylation sites using Net-N-glyc v1.0 [28] and Net-O-glyc v4.0 [29], respectively. The phylogenetic tree was constructed based on both 1052 nts of the S gene and 837 nts of the N gene. Phylogenetic analysis was performed using the maximum likelihood (ML) method of MEGA v7.0 software with branch support and was assessed by 1000 bootstrap resampling iterations.

## 3. Results

### 3.1. Prevalence of Human CoVs

Detection of the four seasonal HCoVs was attempted in the viral RNA extracts of 361 newly collected and archival clinical samples using a one-step RT-PCR assay (Table 2). Of the 361 samples, 21 (5.8%) were positive for HCoVs. HCoV-NL63 and HCoV-HKU1 were not detected in the four winter seasons. Among the studied winter seasons, HCoVs were more prevalent in the winter season of 2015/2016 (20.2%) followed by 2014/2015 (3.2%). No positive samples were detected in the winter season of 2019/2020, and only one positive sample (2%) was detected in 2021/2022. The most detected HCoVs was the OC43 (4.15%), particularly in the winter season of 2015/2016, recording 13.48% of the collected samples (*n* = 89). Two samples were reported to be co-infected with OC43 and 229E (Table 2).

### 3.2. Sequence Analysis and Glycosylation Profiles of S and N Genes

Multiple sequence alignment of the two Saudi HCoV-OC43s strains with international strains allowed recording several mutation sites at the nucleotide and amino acid levels. Among the HCoVs included in this study, the overall nucleotide sequence homology ranged from 94.2% to 99.9%. A total of 43 mutations were recorded in the S gene of strain Riyadh-65-2016 and 38 mutations in Riyadh-05-2022. Of these mutations, 23 changed their corresponding amino acids in strain Riyadh-65-2016 and 22 amino acids have been changed in strain Riyadh-5-2022 (Table 3). Three sequence deletions were reported in strain Riyadh-65-2016: (i) one nucleotide deletion at 352; (ii) two-nucleotide deletion at positions 356 and 357; and (iii) 12 nucleotides deletion at positions 798 to 809. The first two deletions were reported in all strains, including Riyadh-5-2022, except strains from Cote d’Ivoire (MG977445 and MG977447). The 12 nucleotide-deletion was reported in strains of human CoVs from the USA, France, and China and all BCoVs.

**Figure 1:**
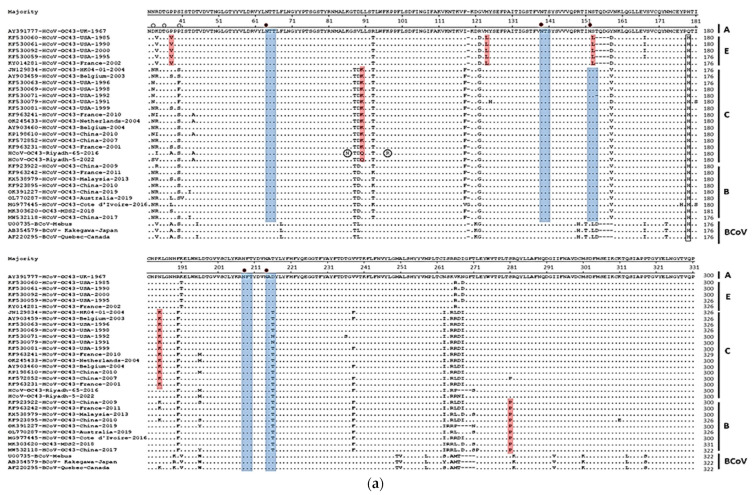

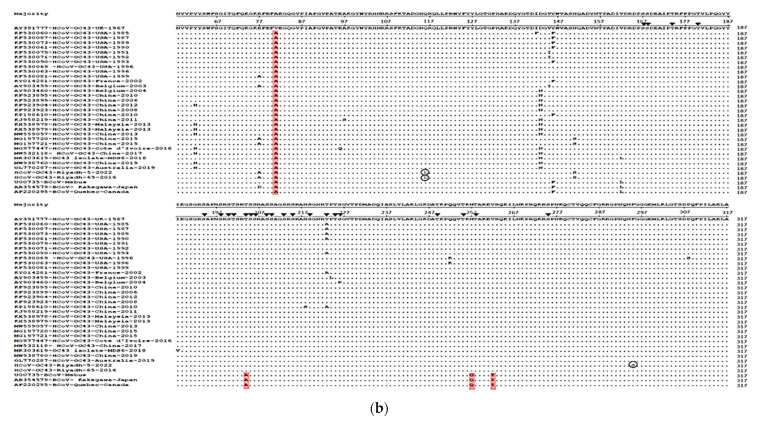
Deduced amino acid alignments of the glomerular (**a**) S gene and (**b**) N gene. Strains that represent the four genotypes were selected, and the alignment was performed by Clustal W (MegAlign program, DNAstar). The strain HCoV-OC43 (accession number: AY391777) isolated during the year 1967 was set as the consensus sequence. Genotype-specific amino acids are highlighted in red. Predicted N-glycosylation sites are highlighted in blue and O-glycosylation sites are indicated by small filled triangles.

At the amino acid level, analysis of the glomerular part of the S protein (S1 subunit) revealed a large number of mutations (Table 3, Figure 1a). Some amino acid changes are characteristic for each genotype. Genotype E strains have a four-codon deletion at positions 153, 154, 155, and 156. The same codon deletion was reported in BCoVs. Strains of genotype E have also characteristic amino acid changes at positions P38V, M123L, and S152L. The majority of genotype C strains have a characteristic amino acid change at position L90K and N180K. Genotype B strains have a characteristic amino acid change at position S276P. Two characteristic amino acid changes were reported for strain Riyadh-65-2016 at positions K86N and K97R. Both Saudi strains have characteristic amino acid change at position L90Q. Four-codons deletion was also reported in strain Riyadh-65-2016 at positions 262, 263, 264, and 265. The same four-codons deletion was reported in strains of BCoVs and China strains of human CoVs. One amino acid change at position Q174H was reported in all strains except for the reference strain AY391777. Glycosylation profiles were assessed by determining the potential N- and O-linked glycosylation sites. The four common N-glycosylation sites in all HCoV-OC43s including Saudi strains are 64N-138N,151N, 207N, and 213N. The pattern of O-glycosylation is not fixed. The potential sites for O-glycosylation vary among the strains through the amino acid residues 33, 36, 37, 38, and 40 (G-score 0.6–0.99).

For the N gene, 10 and 8 mutations were reported in Riyadh-5-2022 and Riyadh-65-2016, respectively. Among these, four mutations in Riyadh-65-2016 and five in Riyadh-5-2022 changed their corresponding amino acids (Table 3). No sequence gaps and/or deletions were reported in the N gene fragment. At the amino acid level, one signature amino acid change (V81A) was observed in all strains except for the reference strain AY391777 (Figure 1b). One characteristic amino acid change (Q116L) was observed in strains of Riyadh-65-2016 and Riyadh-5-2022. One additional amino acid change was reported only in strain Riyadh-5-2022. No characteristic amino acid changes were reported among strains from different countries or different years. Stains of BCoVs have three characteristic amino acid changes at S147A, H200Q, and V205I (Figure 1b). No amino acid residues were predicted as potential sites for N-glycosylation. However, several serine and threonine residues were predicted as potential O-glycosylation sites (G-score 0.5–0.98); 167S, 168S, 174T, 180T, 194S, 198S, 200T, 201S, 202S, 204T, 205S, 206S, 209S, 210S, 213S, 214S, 219S, 223T, 225T, 226S, 249T, 255T, 258T, and 275S.

### 3.3. Phylogenetic Analysis of S and N Genes 

**Figure 2:**
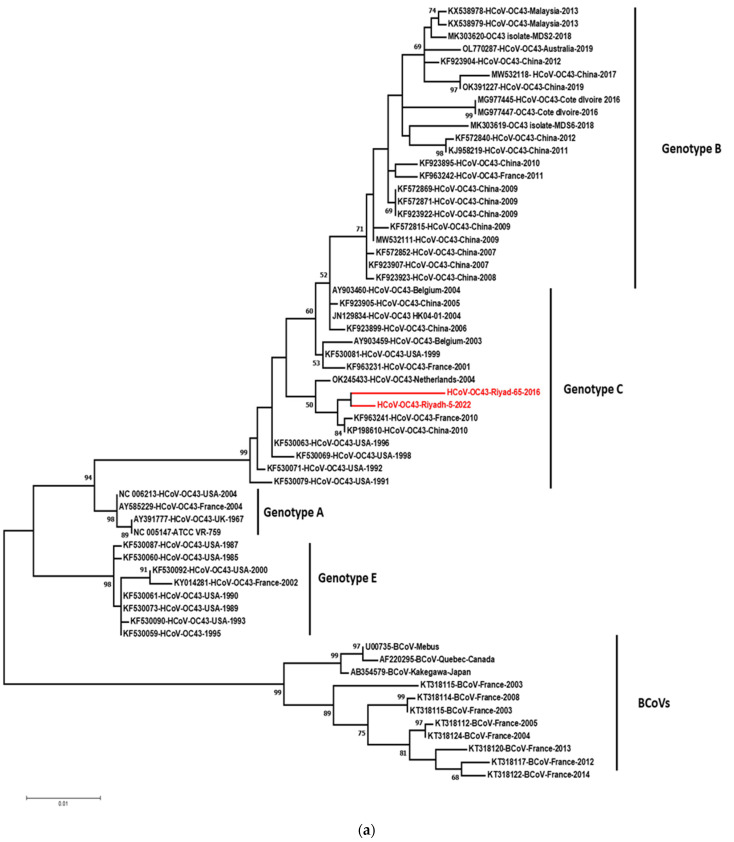

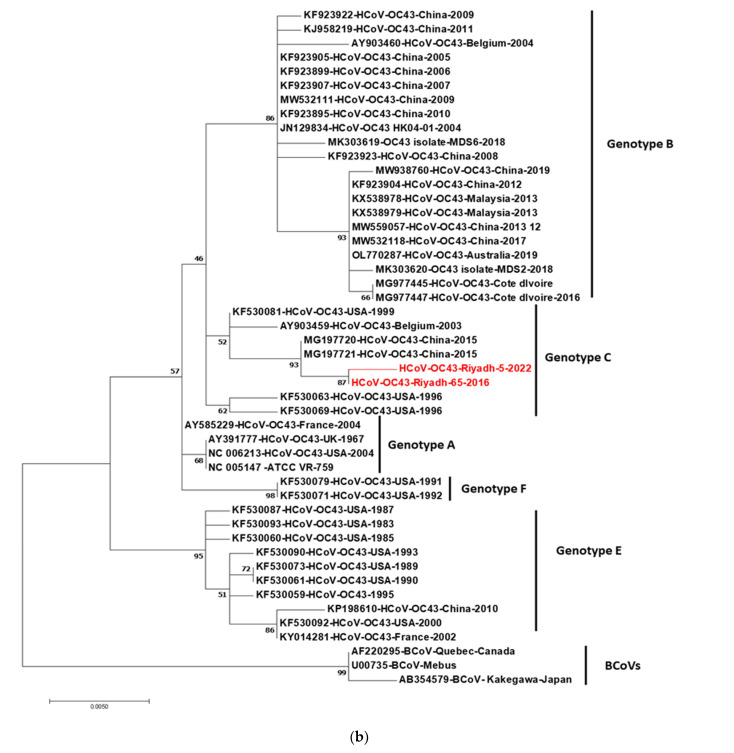
Phylogenetic analysis tress based on (**a**) 1052 nucleotides of the S gene (subunit 1) and (**b**) 837 nt of the N gene. Multiple sequence alignment was performed using Clustal W, and the phylogram was generated by the MEGA 7 program using the maximum likelihood method. Strains of bovine coronaviruses were used to root the trees. Strains in red fonts refer to Saudi stains identified and sequenced in the currents study. Only bootstrap values exceeding 50% are displayed. Saudi strains of the seasons 2015/2016 and 2021/2022 are presented in red font. Genotypes are indicated at the periphery of the phylogram.

The phylogenetic trees constructed based on S and N genes are shown in Figure 2. For the S gene-based tree (Figure 2a), five clusters can be differentiated from the outlier strains of BCoVs. The clustering is supported by high bootstrap values that ranged from 52 to 99. The cluster of BCoVs is divided into two clades where sequences of Mebus, Kakegawa, and Quebec are in one clade and other BCoVs from France in the other subclade. We followed the nomenclature of genotypes adopted by [27,30]. Genotype A included the HCoV-OC43s strains isolated during the year 2004 and the reference strain (AY391777) isolated in 1967. Genotype C included our Saudi strains (Riyadh-65-2016 and Riyadh-5-2022) and strains from the USA, China and France, Belgium, and Netherland. Strains with four-codon deletion (positions: 153, 154, 155, and 156) were grouped into genotype E. Genotype B included the majority of Chinese strains. The tree was constructed based on the N gene six clusters including the cluster of BCoVs (Figure 2b). The clusters of human CoVs were named genotypes A, B, C, E, and F. With few exceptions, these genotypes included the same strains as in the S tree. Some strains showed recombinant genotypes, such as HCoV-OC43-Belgium-2003 (AY903459) and HK04-01-2004 (JN129834) (genotype BC), whereas strains of genotype A (AY391777, NC005147, and NC006213) showed non-recombinant genotype AA. Similarly, strains of genotype E showed a non-recombinant genotype EE. Strains from the USA (KF530079-HCoV-OC43-USA-1991, KF530071-HCoV-OC43-USA-1992) that were grouped (C) into genotype in the S gene tree formed a distinct clade (F) that root genotypes A, B, C.

## 4. Discussion

HCoVs have long been recognized as the commonest cause of respiratory tract infections with a wide range of clinical outcomes [14,31]. The epidemiologies of the four common HCoVs (OC43, 229E, NL63, and HKU1) and their evolutionary kinetics are poorly studied worldwide and particularly in Saudi Arabia. In our study, the prevalence of the four HCoVs was investigated during the four winter seasons. Generally, HCoVs predominate during the winter seasons between December and April with low or no detection frequency in the summer months [13,32]. NL63 is an exception where sporadic cases during the summer were reported [32]. Among the four circulating HCoVs, HCoV-OC43 and HCoV-NL63 are the most prevalent and usually encountered during early childhood [12]. The prevalence of HCoV in the current study was 5.8%, and OC43 was the most frequently detected virus (4.15%) followed by 229E (1.1%). The prevalence pattern in our study in agreement with previous studies from China, the UK, and France [26,32,33,34]. In China, the prevalence of HCoVs among 13,048 tested samples was 2.25% (*n* = 294) during the winter seasons of 2010 to 2015. Of the seasonal HCoVs detected, OC43 was the most prevalent with 60.20% followed by 229E (16.67%), NL63 (14.97%), and HKU1 (7.82%) [14].

In contrast, other studies reported the prevalence of other HCOVs rather than OC43. In Japan, in a 4-year study (2010 to 2013), the prevalence of HCoV in 4,342 samples was 7.6% (*n* = 332) with NL63 recording (3.1%), HKU1 (1.9%), OC43 (1.8%), and 229E (0.9%) [13]. Chow et al., reported the prevalence of HKU1 (64%) in congregate homeless shelter settings followed by NL63 (24%) [11]. In the UK, Gaunt et al. reported the prevalence of HCoV-OC43 in two winter seasons, whereas NL63 predominated in one season [32]. NL63 coronaviruses was the most common coronavirus identified in Michigan, USA [35]. We were unable to detect HCoVs in the tested samples (*n* = 100) collected during the winter season of 2019/2020. This could be attributed to the control measures (i.e., lockdown, traveling restrictions, wearing masks, etc.…) launched by the government to contain the COVID-19 pandemic. During the COVID-19 pandemic, several countries reported disturbances in respiratory virus dynamics [36,37,38]. In South Korea, the positivity rates of eight respiratory viruses (influenza virus, adenovirus, bocavirus, rhinovirus, metapneumovirus, parainfluenza virus, respiratory syncytial virus, and HCoVs) decreased greatly in the year 2020 in comparison with the previous year [39]. In Canada, the positivity rates of influenza A and B, RSV, and enterovirus/rhinovirus decreased dramatically during the year 2020/2021 [40].

To understand the evolutionary kinetics and molecular epidemiology of OC43 in Saudi Arabia, a partial sequence of the S and N genes of two samples, one from the year 2016 and the other sample collected during the year 2022, was constructed. Due to the long period of storage of archived samples and possible degeneration of viral RNA, the sequence of S and N genes was only retrieved from one sample, Riyadh-65-2016. The first attempt to analyze OC43 strains based on the S gene revealed a probable spatial and temporal distribution of genetic clusters [41]. Subsequently, Lau et al., were the first to define genotypes of OC43 stains based on the complete sequence of RdRp, S, and N genes [30]. In the study of Lau et al., OC43s were grouped into four genotypes. Genotype A included the prototype strain VR759, genotypes B and C included contemporary circulating strains, and a recombinant genotype D is B/C genotype [30]. Similarly, Kin et al., have sequenced complete RdRP, S, and N genes of OC43s and compared them to sequences from the USA, Belgium, and Hong Kong. They followed the nomenclature of genotypes established by Lau et al. and the same genotypes were designated. However, they defined a new cluster E which is characterized by the deletion of 12 nucleotides in the S (subunit 1) gene [27]. The impact of such deletion on virus binding and the possibility of cross-species transmission from cattle have been discussed intensively in the study [27]. Genotype E was reported to originate due to natural recombination between the three genotypes: A, B, and C [34]. Two years later, two additional genotypes, F and G, were reported in Malaysia [42]. Reporting such new genotypes in different countries refers to the continuous evolution of HCoV-OC43.

In our study, partial sequences of the S (1052 nt) and N (837 nt) genes were compared with the corresponding international sequences of 59 and 46 OC43s, respectively. The use of partial sequences of S1 (557 nt) and N (558) gens in sequence and phylogeny of OC43s was reported [43]. Phylogenetic trees based on different genes of the same virus can be used to identify recombinant virus strains [15,30,44]. Based on the S and N genes, we have observed four genotypes (A, B, C, and E) with strains Riyadh-65-2016, and Riaydh-5-2022 were grouped into non-recombinant genotype C. The strain Riyadh-65-2016 has a 12-nucleotides deletion which resulted in 4-codons deletions (266K, 267N, 268G, 269F). Such four-codons deletion in the glomerular part of the S1 subunit may affect virus binding to host cellular receptors. Interestingly, all BCoVs, strains isolated from the USA (2004) (NC006213), and strains isolated from France (2004) (AY585229) have the same four-codons deletion. This finding supports that Riyadh-65-2016 originated as a recombination with cattle and crossed species boundaries.

## 5. Conclusions

In conclusion, the current study reported the prevalence of the four common HCoVs in Riyadh, Saudi Arabia. OC43 was the most frequently detected followed by NL63, whereas the other two HCoVs, HKU1 and 229E, were not reported in the four winter seasons. Due to control measurements applied by the government, none of the common HCoVs was detected in the winter season of 2019/2020. Sequence and phylogenetic analysis data of OC43 were based on partial sequences of S and N genes. Four genotypes were observed in the S-based tree with Saudi strains grouped into the genotype C. Nucleotide, and amino acid sequences of the S gene revealed many characteristics and signature amino acids for each genotype. Interestingly, strain Riyadh-65-2016 shared sequence similarity and four-codons deletion with BCoVs which suggest recombination events for its origin. It is recommended to perform long-term surveillance and analyze the whole OC43 genomes which could reveal the recombination patterns and give more insights into the evolutionary dynamics of HCoV-OC43.

## Figures and Tables

**Table 1:** Oligonucleotide primers used in the study for detection, typing, and sequencing of seasonal coronaviruses.

Primer Name	Sequence (5′–3′)	Amplicon Size (bp)	Ref
Detection of CoVs (RdRp gene)			
panCoV-F	AARTTYTAYGGHGGYTGG	668	[25]
panCoV-R	GARCARAATTCATGHGGDCC		
Typing primers (RdRp gene)			
HCoV-OC43			
OC43-F	CTGGGATGATATGTTACGCCG	444	[26]
OC43-R	TATTCTGTGACAAAGGTTG		
HCoV-229E			
229E-F	GTGTGATAGAGCTATGCCCTCA	463	
229E-R	GTAACCAAGTCCAGCATAAGTT		[26]
HCoV-NL63			
NL63-F	AATAATATGTTGCGTACTTTA	472	
NL63-R	TCATTGAAAAATGTTTCCTA		[26]
HCoV-HKU1			
HKU1-F	AAAGGATGTTGACAACCCTGTT	453	
HKU1-R	ATCATCATACTAAAATGCTTACA		[26]
Sequencing primers			
OC43-SF	CCA ATG GCT TTT GCT GTT ATA GGA G	1525	This study
OC43-SR	GTA CCT GCA GGA CAA GTG CC		
OC43NF	CAGCAACCATCAGGAGGGAA	891	This study
OC43NR	AAACATCCTTCTGGGGCTG		

**Table 2:** The prevalence of the four common HCoVs in the studies of winter seasons.

Winter Season	Total No. of Samples	No. of Positive Samples	Human CoVs				
			OC43	229E	NL63	HKU1	Mixed
2014–2015	122	4 (3.2%)	2 (1.64%)	2 (1.64%)	0	0	0
2015–2016	89	18 (20.2%)	12 (13.5%)	2 (2.25%)	0	0	2 (2.25%)
2019–2020	100	0	0	0	0	0	0
2021–2022	50	1 (2%)	1 (2%)	0	0	0	0
Total	361	21 (5.8%)	15 (4.15%)	4 (1.1%)	0	0	2 (0.55%)

**Table 3:** Mutation record of nucleotides and amino acids sequences.

Genome Location	Riyadh-65-2016		Genome Location	Riyadh-5-2022	
	Nt Sequence	Amino Acid		Nt Sequence	Amino Acid
The S gene/protein					
	A101T	K34I		A98G	D33S
	C112G	P38A		A101T	K34V
	C118T	P40S		C118T	P40S
	T131C	D44A		T131C	D44A
	G258C	K86N		G263C	S88T
	G263C	S88T		A267C	V89D
	T266A	V89D		T269A	L90Q
	T269A	L90Q		C278G	R93T
	G278C	R93T		A352T	I118F
	A290G	K97R		C361G	R121G
	A352T	I118F		A470T	Y157V
	C361G	R121G		A534T	Q178H
	A470T	Y157V		G566T	R189F
	A534T	Q178H		T583A	L195M
	G566T	R189F		A644C	D215T
	T583A	L195M		A785T	N262I
	A644C	D215T		G792C	K264R
	G710T	V237F		G798T	K266N
	A785T	N262I		A800T	N267I
	G792C	K264R		C809G	T270S
	T793G	V265P		T1016C	L339P
	T1016C	L339P		A1018G	N340D
	A1018G	N340D			
The N gene/protein					
	A230C	E77A		A230C	E77A
	T242C	V81A		T242C	V81A
	A347T	Q116L		A347T	Q116L
	A452G	N151S		A452G	N151S
				T884C	F295S

## Data Availability

All data generated or analyzed during this study are included in this published article.
